# Deep Eutectic Electrolytes for Lithium Metal Batteries: A Review

**DOI:** 10.1002/advs.202517939

**Published:** 2025-11-21

**Authors:** Di‐Chen Wu, Xi‐Long Wang, Shi‐Jie Yang, Yao‐Hui Zhu, Zi‐Hao Zuo, Zheng Liao, Jia Liu, Hong Yuan, Jia‐Qi Huang

**Affiliations:** ^1^ School of Interdisciplinary Science Beijing Institute of Technology Zhuhai 519088 China; ^2^ School of Interdisciplinary Science Beijing Institute of Technology Beijing 100081 China; ^3^ Beijing Key Laboratory of Lignocellulosic Chemistry College of Materials Science and Technology Beijing Forestry University Beijing 100083 China

**Keywords:** deep eutectic electrolytes, liquid electrolytes, lithium metal batteries, solid‐state electrolytes

## Abstract

Deep eutectic electrolytes (DEEs) have emerged as highly promising materials for next‐generation lithium (Li) metal batteries (LMBs), owing to the high ionic conductivity, excellent thermal stability, intrinsic flame retardance, and wide electrochemical stability window. Although the research of DEEs in LMBs is expanding rapidly, in‐depth understanding of DEEs is still in their infancy. This review systematically examines the formation mechanism, classification, fundamental properties, and applications of DEEs across both liquid and solid‐state LMBs. It highlights the technical advantages of DEEs in addressing critical issues such as ionic conduction, interfacial stabilization, flame retardance, and battery safety. Furthermore, future research directions are proposed, aiming at overcoming current limitations of conventional electrolytes in energy storage technology and promoting the development and practical applications of DEEs in advanced LMBs with high safe and energy density.

## Graphical abstract

**Deep eutectic electrolytes** (DEEs) promise next‐generation high‐energy‐density lithium (Li) metal batteries owing to their high ionic conductivity and superior safety. This review systematically summarizes the formation mechanism, properties, and application of DEEs in interface stabilization, while also outlining future directions to guide the development of high‐energy, safer Li metal batteries.

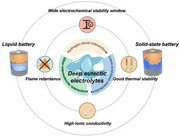

## 1 Introduction

The global transition to clean energy systems has intensified the demand for next‐generation energy storage technologies with high energy density, intrinsic safety, and environmental sustainability.^[^ ^1^, ^2^, ^3^, ^4^ ^]^ While conventional lithium (Li)‐ion batteries (LIBs) currently dominate the energy storage landscape, the electrolytes, composed of flammable organic components and thermally unstable Li salts (e.g., lithium hexafluorophosphate (LiPF_6_)), pose fundamental limitations.^[^ ^5^, ^6^, ^7^, ^8^ ^]^ The traditional carbonate‐based electrolytes are generally incompatible with high‐voltage cathodes (>4.5 V) and Li metal anodes, leading to the electrode/electrolyte interface degradation and rapid capacity decay.^[^ ^9^, ^10^, ^11^, ^12^, ^13^ ^]^ Moreover, the flammability of electrolyte systems exhibits severe safety risks including combustion and even explosion.^[^ ^14^, ^15^, ^16^ ^]^ These challenges underscore the urgent need for novel electrolyte systems that simultaneously achieve a wide electrochemical stability window (ESW) and non‐flammability to accommodate next‐generation high‐energy‐density Li metal batteries (LMBs).^[^ ^17^, ^18^, ^19^, ^20^, ^21^ ^]^

Deep eutectic electrolytes (DEEs) as a transformative class of designable solvents, have sparked extensive research interest owing to a unique combination of properties (e.g., low melting point, good thermal stability, and flame retardance) to address conventional electrolyte limitations.^[^ ^22^, ^23^ ^]^ Structurally, DEEs form through hydrogen bond interactions between hydrogen bond acceptors (HBAs) and hydrogen bond donors (HBDs),^[^ ^24^ ^]^ creating a highly tunable molecular architecture. Strong hydrogen bond interactions can effectively disrupt the regular structure arrangement of solvent molecules, thereby lowering the melting point of the DEEs.^[^ ^25^, ^26^ ^]^ Meanwhile, these interactions can spatially confine the free solvent molecules within formed hydrogen bond networks, reducing the vapor pressure of the solvent molecules and improving the thermal and electrochemical stability of electrolytes.^[^ ^27^, ^28^ ^]^ Consequently, these attributes position DEEs as ideal electrolyte candidates used in high‐voltage LMBs with high energy density and high safety.^[^ ^29^, ^30^, ^31^ ^]^

Despite promising demonstrations in prototype LMBs, the widespread adoption of DEEs faces several fundamental and practical challenges rooted in their unique chemical structure.^[^ ^32^, ^33^ ^]^ The strong hydrogen bond interactions between HBAs and HBDs, which is beneficial for thermal stability and non‐flammability, create a molecular environment that inherently restricts Li⁺ mobility. The confined solvent molecules within the rigid hydrogen bond network impose high energy barriers for the migration of solvated Li^+^, typically leading to a reduced room‐temperature Li^+^ conductivities.^[^ ^34^, ^35^, ^36^ ^]^ Moreover, the persistent hydrogen bond activity generally interferes the interface degradation between Li metal anode and electrolyte, leading to continuous electrolyte reduction and dendritic growth at Li anode surfaces.^[^ ^37^ ^]^ The stringent requirements for electrochemical stability severely limit viable HBAs/HBDs combinations. These challenges stem from an incomplete mechanistic understanding of DEEs. Therefore, the fundamental and in‐depth understanding of DEEs will be critical for designing next‐generation DEEs that overcome current performance bottlenecks in practical LMB systems.

**Figure 1:**
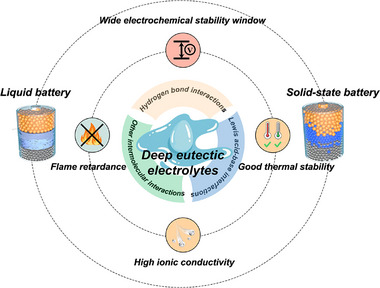
Key properties and applications of DEEs in LMBs.

In this review, we comprehensively summarize recent advancements of DEEs, with particular emphasis on electrode/electrolyte interfacial stability in LMBs (**Figure** **1**). Beginning with the introduction of the fundamental physicochemical characteristics of DEEs, we systematically analyze the critical structure‐property relationships in DEEs systems. Then, the practical implementation of DEEs in both conventional liquid and emerging solid‐state LMBs are examined, highlighting key performance metrics and interfacial compatibility. Finally, the future perspectives on developing high‐performance DEEs for high‐energy‐density LMBs are included. In summary, this work aims to establish a materials innovation roadmap to guide the rational design of high‐performance DEEs that meet the increasingly requirements of future energy storage systems.

## 2 Fundamentals of DEEs

DEEs represent a class of emerging electrolytes with a low melting point formed through complexation between HBDs and HBAs via non‐covalent intermolecular interactions.^[^ ^38^, ^39^ ^]^ The core characteristic of DEEs is that their melting point is significantly lower than that of their individual components, and they often maintain liquid states at ambient conditions.^[^ ^40^, ^41^, ^42^ ^]^ This unique phase behavior stems from the disruption of crystalline lattices through intermolecular interactions, enabling tunable physicochemical properties through rational selection of HBAs/HBDs combinations and molar ratios.^[^ ^43^, ^44^, ^45^ ^]^ The modular design endows DEEs with distinct advantages in Li^+^ conduction, electrochemical stability, thermal stability, and flame retardance, thereby contributing to replacing conventional organic electrolytes in electrochemical applications.^[^ ^45^, ^46^, ^47^ ^]^ To establish a consistent vocabulary and enhance the readability of this review, **Table** **1** compiles the key terminology, abbreviations, and definitions for deep eutectic systems.

**Table 1:** Definition box.

Full name	Abbreviation	Definition
Hydrogen bond donors	HBDs	The atom or functional group that provides the hydrogen atom for the formation of a hydrogen bond.
Hydrogen bond acceptors	HBAs	The atom or functional group that provides the electron‐rich site for the formation of a hydrogen bond with an electron‐deficient hydrogen.
Deep eutectic solvents	DESs	A class of multicomponent eutectic liquid systems characterized by a significant depression in melting point relative to the melting points of their individual components, formed by mixing HBAs and HBDs.
Deep eutectic electrolytes	DEEs	A functional class of DESs incorporating Li salts that function as the ion‐conducting electrolyte in electrochemical devices.

### 2.1 Formation Mechanism of DEEs

**Figure 2:**
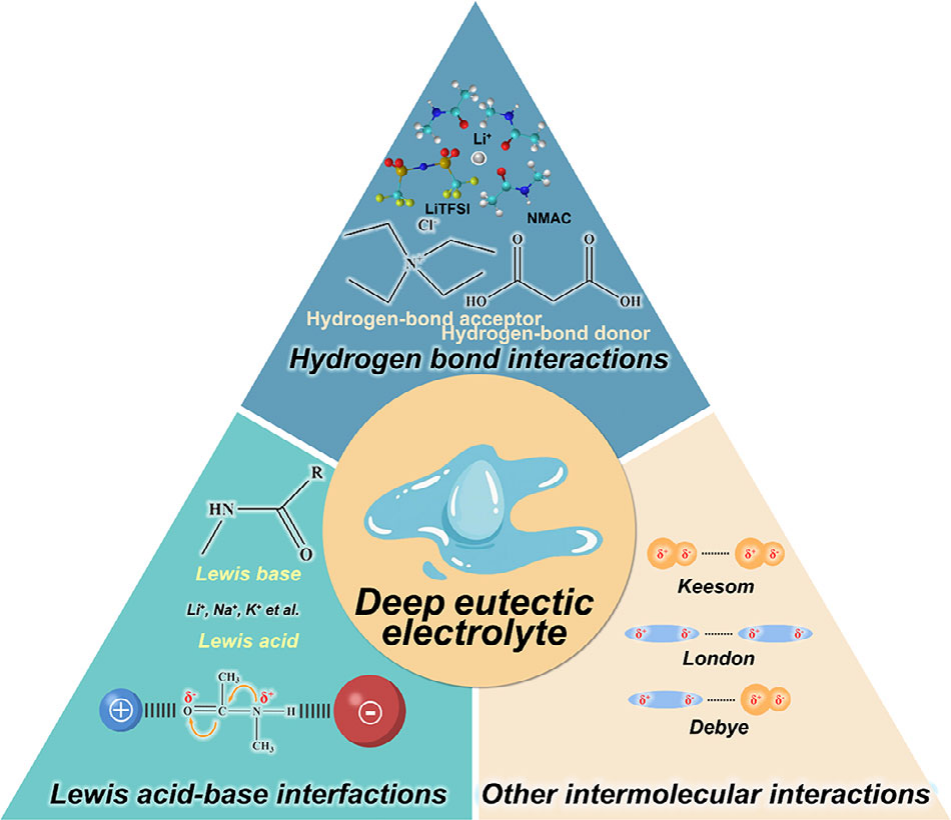
Formation mechanisms of DEEs.

DEEs are typically synthesized through the thermal heating or mechanical grinding of HBAs and HBDs.^[^ ^45^, ^48^, ^49^ ^]^ The defining feature of these systems is their markedly depressed eutectic point compared to the melting temperatures of individual components, often achieving liquid states at room temperature despite containing solid precursors. This unique phase behavior is attributed to three fundamental interaction mechanisms that govern the formation of DEEs (**Figure** **2**).

#### 2.1.1 Hydrogen Bond Interactions

The predominant mechanism involves strong hydrogen bond interactions between HBAs (e.g., choline chloride (ChCl), metal halides) and HBDs (e.g., amides, alcohols, and carboxylic acids),^[^ ^50^, ^51^, ^52^ ^]^ which significantly induces charge delocalization. Charge delocalization can effectively weaken the intermolecular forces within each component, thereby significantly reducing the eutectic point of the mixture compared to pure constituents.^[^ ^40^ ^]^ The directional nature of hydrogen bonds facilitates the self‐assembly of ordered solvation structures critical for ionic transport.

#### 2.1.2 Lewis Acid‐Base Interactions

Following Pearson's hard‐soft acid‐base theory, alkali metal cations (e.g., Li⁺, Na⁺) in electrolyte salts act as Lewis acids coordinating with electron‐donating groups (e.g., amides, carbonyl, ether) of Lewis base species.^[^ ^53^, ^54^ ^]^ The strengths of these Lewis acid‐base interactions range from 4 to 100 kJ mol^−1^, sufficient to modify coordination environments and thereby generate a typical DEEs.^[^ ^55^ ^]^ These Lewis acid‐base interactions without covalent bond formation effectively reduce the melting point of DEEs. Li salt‐based DEE systems (e.g., lithium bis((trifluoromethyl)sulfonyl)azanide (LiTFSI)‐urea, lithium nitrate (LiNO_3_)‐acetamide) demonstrate particular relevance for battery electrolytes.^[^ ^56^ ^]^

#### 2.1.3 Van der Waals Interactions

Van der Waals interactions encompass dipole‐dipole (Keesom), dipole‐induced dipole (Debye), and dispersion (London) forces. As the weakest class of non‐covalent interactions, their binding energies range from 0.4 to 4 kJ mol^−1^. Despite their modest strength, these ubiquitous forces operate between all two molecular species and play a crucial role in the structural stabilization of DEEs through their collective contributions.^[^ ^57^ ^]^ Also, van der Waals interactions have an influence on bulk physicochemical properties and modulate the strength of hydrogen bonds and Lewis acid‐base interactions, thereby contributing to the unique solvent behavior of DEEs.^[^ ^58^ ^]^

Overall, the formation and properties of DEEs are precisely regulated through the synergistic interplay of hydrogen bonds, Lewis acid‐base interactions, and van der Waals forces. These interactions do not operate in isolation but collectively govern the eutectic process as an integrated combination. Hydrogen bonds provide directional cohesion, Lewis acid‐base interactions modulate the local coordination environment, and van der Waals forces contribute to structural stabilization through delocalized intermolecular attraction. The relative strength and synergistic effects of these interactions determine the disruption of the native stable structure of individual component and the successful assembly of the eutectic system. Consequently, their balance serves as a fundamental design principle for tailoring DEEs formation and functionality.

### 2.2 Classification of DEEs

Abbott and his colleagues made groundbreaking contributions to the discovery and development of deep eutectic solvents (DESs) and laid the foundation for the field of DEEs. In 2003, Abbott et al.^[^ ^59^ ^]^ first introduced the term “DES” to describe the mixture of ChCl and urea, which exhibited a significantly lower melting point than its individual components. Over the past two decades, the DESs system has been further expanded with the recognition of numerous potential components, leading to the inclusion of various DESs formulations.

Subsequently, Abbott et al.^[^ ^60^ ^]^ proposed the most widely used classification method for DESs, categorizing them based on their HBDs and HBAs in the general formula Cat^+^X^−^zY. In the standard formula, Cat^+^ represents an ammonium, phosphonium, or sulfonium cation; X^−^ is a Lewis base; Y is a Brønsted acid; and z denotes the number of Y molecules interacting with the anion. Based on this framework, DESs can be classified into four primary types (**Table** **2**). Type I consists of metal halides and quaternary ammonium salts; Type II is formed by replacing anhydrous metal halides with hydrated metal halides and combining them with quaternary ammonium salts; Type III forms via hydrogen bond interactions between HBDs and quaternary ammonium salts; Type IV consists of HBDs and metal halides. Notably, Abranches et al.^[^ ^61^ ^]^ recently identified a fifth type (Type V), which is composed exclusively of non‐ionic HBDs and HBAs molecules. Unlike other DESs, Type V systems lack ionic components but still exhibit characteristic of DESs properties, such as depressed melting points, primarily due to strong hydrogen bond interactions.

**Table 2:** Classification of DESs.

Type	Formula	Composition	Example	Refs.
I	Cat^+^X^−^zMCl_x_ M = Zn, Sn, Fe, Al, Ga, In	metal halides + quaternary ammonium salts	ZnCl_2_ + ChCl	[65]
II	Cat^+^X^−^zMCl_x_yH2O M = Cr, Co, Cu, Ni, Fe	hydrated metal halides + quaternary ammonium salts	Zn(ClO_4_)_2_·6H_2_O + Succinonitrile	[66]
III	Cat^+^X^−^zRZ Z = CONH_2_, COOH, OH	HBD + quaternary ammonium salts	ChCl + Urea	[59]
IV	MCl_x_ + RZ = MCl+ x‐1∙RZ+MCl_x+1_ M = Al, Zn Z = CONH_2_, OH	HBD + metal halides	LiTFSl + N‐methylacetamide	[45]

Notably, DEEs, a specialized subclass of DESs incorporating metal salt, have garnered significant interest due to their unique properties. DEEs can be further categorized into two types. One type is formed by dissolving metal salts in DESs,^[^ ^62^, ^63^ ^]^ while the otheris directly formed by HBDs and metal salts. As analogs to ionic liquids (ILs), DEEs demonstrate flame retardance, excellent stability, high ionic conductivity, and wide ESW. In particular, DEEs based on Li salts (e.g., LiTFSI, LiNO_3_, and LiPF_6_) combined with HBDs have been explored for applications in LMBs.^[^ ^64^ ^]^

## 3 Properties of DEEs

The core characteristic of DEEs lies in their highly designable physicochemical properties. Specifically, these properties can be precisely tailored through rational component selection and stoichiometric control, which endows DEEs a distinct advantage to meet diverse application requirements.^[^ ^67^, ^68^ ^]^ Considering the application of DEEs in LMBs, a deep understanding of fundamental physicochemical properties is essential for elucidating their operational mechanisms and electrochemical performance.

### 3.1 High Ionic Conductivity

Ionic conductivity is a key parameter for evaluating the mobility of ions within an electrolyte. DEEs exhibit high ionic conductivity, typically in the range of 10^−2^–10^−3^ S cm^−1^ at room temperature, which is comparable to that of traditional liquid electrolytes.^[^ ^69^ ^]^ In this system, the HBAs (e.g., LiTFSI) spontaneously dissociate into cations (Li^+^) and anions (TFSI^−^), providing a high concentration of charge carrier similar to those in ILs. This establishes a robust material basis for the high conductivity of DEEs. The moderate hydrogen bond network promotes Li salt dissociation and weakens Li⁺‐anion coordination, thereby facilitating Li⁺ mobility.^[^ ^70^ ^]^ However, an excessively strong hydrogen bond interaction can increase the viscosity of DEEs and elevate the energy barrier for Li^+^ diffusion, which thereby impedes Li^+^ conduction.^[^ ^71^ ^]^ Moreover, intense Li^+^‐HBD interactions suppress the vehicular transport of solvated Li^+^. As this is often considered as the dominant Li^+^ transport mechanism in DEEs, its suppression fundamentally limits the enhancement of ionic conductivity.^[^ ^72^ ^]^ Tuning the HBA/HBD molar ratio offers an effective strategy to optimize hydrogen bond density and enhance Li^+^ transport.^[^ ^73^ ^]^ Additionally, the ionic conductivity of DEEs is temperature‐dependent. As temperature rises, reduced viscosity and enhanced ion mobility collectively improve conductivity, which is well‐described by the Vogel‐Fulcher‐Tammann (VFT) equation.^[^ ^74^ ^]^

### 3.2 Wide ESW

DEEs generally possess a wide ESW, rendering them highly suitable for high‐voltage battery applications. In some cases, DEEs even surpass traditional ILs in electrochemical stability. The ESW of DEEs is mainly determined by the chemical properties of the constituents and the intermolecular interactions within the eutectic system.^[^ ^65^ ^]^ The dense hydrogen bond network and charge delocalization effects can significantly help stabilize molecular orbitals and suppress undesirable redox reactions at the electrode interface.^[^ ^75^ ^]^ However, it is worth noting that trace water or other impurities in DEEs can lead to preferential side oxidations (e.g., water electrolysis), which may compromise the ESW and alter the electrolyte structure.^[^ ^76^, ^77^ ^]^

### 3.3 High Thermal Stability

The thermal stability of DEEs is a critical performance indicator in high‐temperature applications. Compared with ILs, DEEs exhibit a unique staged decomposition behavior under heating. The hydrogen bond network in the DEE system disintegrates first under high‐temperature conditions, leading to separation of HBDs and HBAs, followed by volatilization or decomposition of the less stable component (often the HBDs), and finally degradation of the HBAs at elevated temperatures.^[^ ^78^ ^]^ The thermal stability of DEEs is predominantly dictated by the strength and coherence of their intermolecular interactions. Strong hydrogen bond interactions can significantly enhance structural integrity and raise the decomposition onset temperature. Conversely, weak hydrogen bond interactions (e.g., intramolecular hydrogen bonds or low electronegativity of anions) can induce loose network structure, which thereby leads to premature breakdown of these networks and the decomposition of DEEs at high temperatures. In addition, the uniformity of intermolecular interactions also has an important impact on the thermal stability of DEEs. The thermal stability is maximized near the eutectic composition, where the hydrogen bond network is most dense and free from uncomplexed species. Deviation from this eutectic ratio introduces free HBAs or HBDs molecules, which will weaken the overall interaction and reduce the thermal stability.^[^ ^78^ ^]^

### 3.4 Flame Retardance

Liquid combustion requires the fulfillment of the “fire triangle” conditions, that is the simultaneous presence of fuel vapor, oxidant (typically oxygen), and an ignition source. Similar to ILs, DEEs exhibit exceptionally low vapor pressures due to strong intermolecular forces, such as hydrogen bonds and electrostatic interactions.^[^ ^22^ ^]^ For instance, Boisset et al. reported a vapor pressure of only 20 Pa at room temperature for a LiTFSI‐NMAC‐based DEE, which is much lower than that of water (7380 Pa) and acetonitrile (1.02 × 10^5^ Pa).^[^ ^79^ ^]^ The suppressed volatility impedes the formation of flammable vapor, making DEEs inherently non‐flammable. Even under direct flame exposure, DEEs resist ignition and do not support sustained combustion, thereby enhancing battery safety.

To elucidate the performance advantages of DEEs over traditional electrolytes and ILs more clearly and intuitively, **Table** **3** systematically compares their key properties, including ionic conductivity, ESW, and thermal stability, further highlighting their potential for energy storage applications.

**Table 3:** Performance comparison of different electrolytes.

Type	Example	Ionic conductivity at 25 °C [mS cm⁻¹]	ESW (V, vs Li/Li^+^)	Thermal stability [°C]	Refs.
DEEs	LiFSI/prop‑1ene‐1,3‐sultone	1.77	5.1	>150	[80]
	LiTFSI/succinonitrile/butyrolactam	2.83	4.5	>150	[34]
	LiDFOB/succinonitrile/1,3,5‐trioxane	4.1	5.6	/	[81]
	LiTFSI/LiDFOB/succinonitrile	1.26	5	>150	[82]
Traditional electrolytes	1 M LiPF_6_‐EC/DMC	3.22	/	<50	[82]
	1 M LiPF_6_‐DMC	2.6	4.5	<50	[83]
	1 M LiPF6‐EC/EMC	>1	4.2	/	[84]
ILs	LiFSI/1‐ethyl‐3‐methylimidazolium bis(fluorosulfonyl)imide	2.6	4.6	300	[85]
	LiFSI/1‐ethyl‐3‐methylimidazolium bis(trifluoromethylsulfonyl) imide	2.8	5.4	250	[86]
	1‐Methyl‐1‐propyl pyrrolidinium bis(trifluoromethanesulfonyl)imide	4.83	4.75	<100	[87]

## 4 Application of DEEs in Liquid Batteries

Over the past few decades, LMBs have made remarkable advancements in the field of energy storage. The development of electrode materials and electrolytes technology has progressively matured.^[^ ^88^, ^89^, ^90^, ^91^ ^]^ Despite abundant availability, low cost, and excellent electrochemical performance, conventional liquid electrolytes still face inherent limitations.^[^ ^92^, ^93^, ^94^, ^95^ ^]^ These electrolytes are mostly composed of flammable organic solvents. On one hand, these organic solvents are prone to side reactions with Li metal anodes, resulting in a solid electrolyte interface (SEI) that is structurally fragile and chemically unstable. This unstable SEI tends to fracture during cycling, leading to not only the continuous depletion of active Li and electrolyte but also the growth of Li dendrites, seriously deteriorating the interfacial stability.^[^ ^96^, ^97^, ^98^, ^99^ ^]^ On the other hand, conventional electrolytes exhibit poor thermal stability and are susceptible to combustion or explosion under high‐temperature conditions or internal short circuits, seriously threatening the safety and reliability of the battery.^[^ ^100^, ^101^, ^102^, ^103^ ^]^

### 4.1 Improved Interfacial Stability

**Figure 3:**
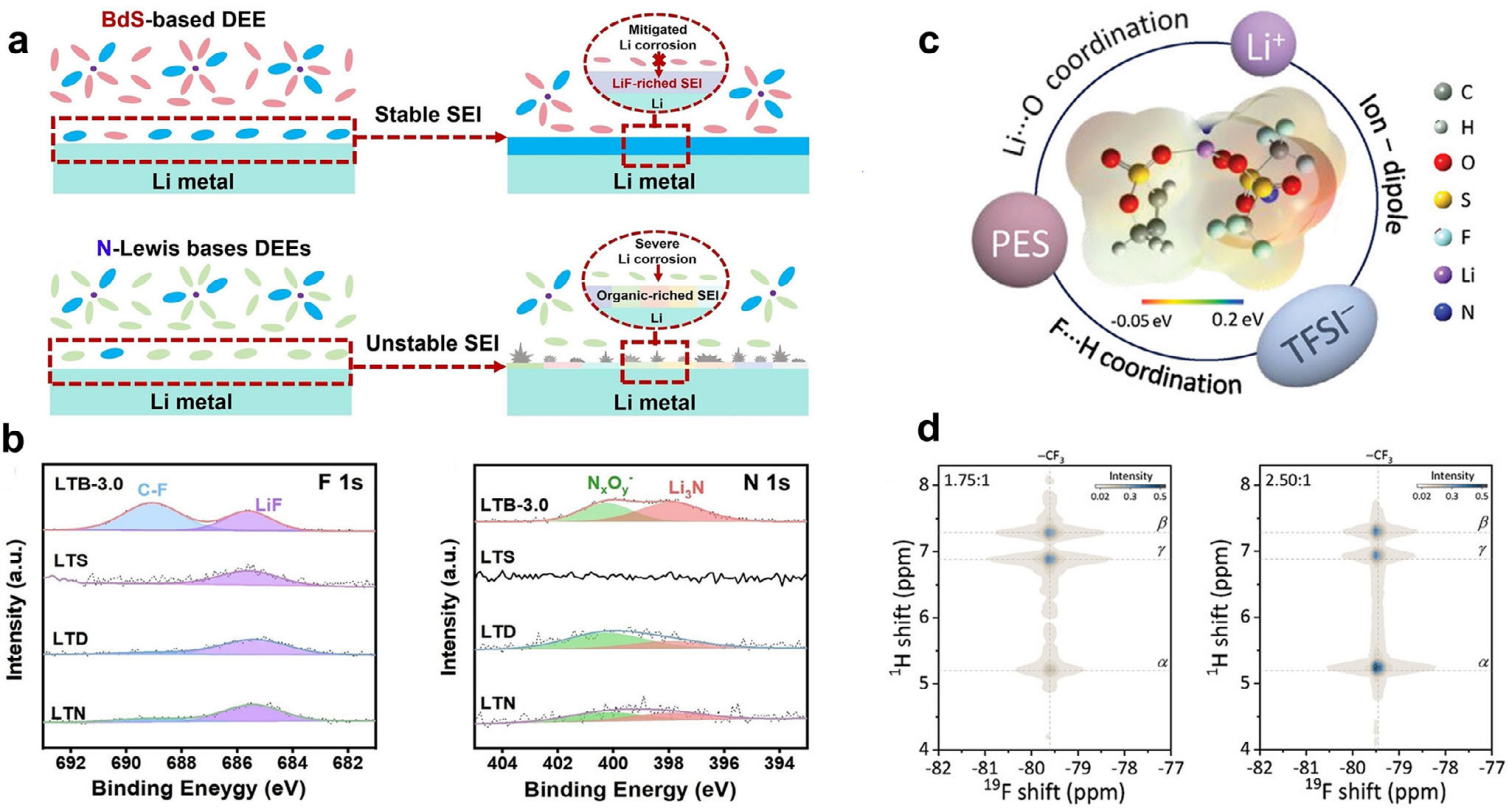
a) Schematic demonstration of the Li compatibility with BdS or typical N‐containing Lewis bases‐based DEE. Reproduced with permission.^[^ ^104^ ^]^ Copyright 2024, Wiley‐VCH. b) The high‐resolution X‐ray photoelectron spectroscopy spectra of the F 1s and N 1s regions. Reproduced with permission.^[^ ^104^ ^]^ Copyright 2024, Wiley‐VCH. c) Schematic illustration and calculated electrostatic potential (ESP) map of the DEE. Reproduced with permission.^[^ ^106^ ^]^ Copyright 2024, Wiley‐VCH. d) 2D ^1^H−^19^F heteronuclear chemical shift correlation (HETCOR) NMR spectra of the DEE at different molar ratios. Reproduced with permission.^[^ ^106^ ^]^ copyright 2024, wiley‐vch.

As mentioned above, one of the biggest challenges faced by LMBs is the interfacial instability, which stems from the intrinsic limitations in the physicochemical properties of the components that make up conventional liquid electrolytes. Recently, DEEs provide a new potential to improve the interfacial stability of LMBs. DEEs facilitate the formation of a robust and compact SEI by modulating the solvation structure of Li^+^, which effectively passivates the Li anode surface and inhibits the growth of Li dendrites.^[^ ^34^ ^]^ For instance, Zhou et al.^[^ ^104^ ^]^ prepared a new DEE by combining solid butadiene sulfone (BdS) and LiTFSI. The low adsorption energy and minimal deformation of BdS on the Li metal surface lead to weak BdS adsorption, thereby promoting preferential LiTFSI decomposition and the formation of a stable LiF‐rich SEI (**Figure** **3**a,b). This DEE exhibited a high ionic conductivity of 1.48 × 10^−3^ S cm^−1^ at room temperature and significantly improved intrinsic safety. In addition, DEEs demonstrate excellent electrochemical stability, which can reduce parasitic side reactions, lower interfacial impedance, and promote uniform Li deposition, thus improving the cycle performance of the battery.^[^ ^105^ ^]^ Ding et al.^[^ ^106^ ^]^ achieved precise control over the local coordination environment of Li⁺ in DEE by synergistic regulation of F···H and Li···O interactions, thereby reducing the migration barriers of Li^+^ and increasing its transport kinetics (Figure 3c). Notably, the enhanced α‐H···F coordination endowed DEE with excellent electrochemical stability (Figure 3d). Interestingly, DEEs can also enhance the solubility of SEI‐forming additives via intermolecular interactions, facilitating the formation of inorganic‐rich interphases that improve deposition homogeneity and anode stability.^[^ ^45^ ^]^ These capabilities highlight the potential of DEEs in enabling long‐term stable operation of LMBs.

### 4.2 High‐Temperature Compatibility

With the rapid development of the new energy industry and the increasing demand for energy storage under extreme environment conditions, there is a pressing need for batteries capable of operating reliably at high temperatures.^[^ ^107^, ^108^, ^109^ ^]^ Under high‐temperature conditions, electrolytes must possess a thermodynamically stable solvation structure, a robust SEI, and high thermal safety.^[^ ^110^, ^111^, ^112^ ^]^ However, traditional liquid electrolytes have significant drawbacks under extremely high‐temperature conditions. The solvent undergoes violent volatilization and decomposition reactions driven by heat, which directly disrupts the dynamic equilibrium of ion transport and thereby results in a rapid decay in ionic conductivity.^[^ ^113^, ^114^, ^115^ ^]^ In addition, these processes also trigger side reactions of electrode materials, which not only accelerate the degradation of battery performance but also cause potential safety hazards.^[^ ^116^, ^117^, ^118^ ^]^

**Figure 4:**
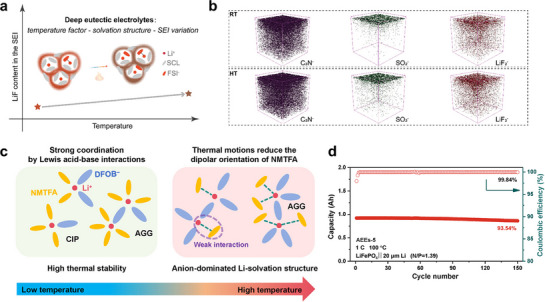
a) Schematic of the temperature‐regulated solvation structure and SEI. Reproduced with permission.^[^ ^122^ ^]^ Copyright 2023, ACS Publications. b) the corresponding 3D views of several typical second ion fragments with the SEI formation temperature of RT and HT. Reproduced with permission.^[^ ^122^ ^]^ Copyright 2023, ACS Publications. c) Schematic diagram of temperature‐dependent solvation structures as a function of temperature. Reproduced with permission.^[^ ^123^ ^]^ Copyright 2024, Elsevier. d) Cycling performance of the 1 Ah LiFePO_4_||Li pouch cell at 1C and 100 °C. Reproduced with permission.^[^ ^123^ ^]^ Copyright 2024, Elsevier.

DEEs offer a promising solution for high‐temperature battery operation. In these systems, all molecules engage in a synergistic network through hydrogen bond interactions, such as Lewis acid‐base interactions and other intermolecular forces, which significantly reduce the fraction of free solvent molecules and thereby greatly expands the working temperature range.^[^ ^60^, ^119^, ^120^ ^]^ ​ Under high‐temperature conditions, DEEs can facilitate the formation of weakly solvated high‐concentration electrolyte structures that promote the development of stable electrode/electrolyte interface.^[^ ^121^ ^]^ Hou et al.^[^ ^122^ ^]^ demonstrated that elevated temperatures can strengthen the interactions between Li⁺ and anions, thereby increasing the content of LiF in the SEI (**Figure** **4**a,b). Similarly, Fang et al.^[^ ^123^ ^]^ observed that thermal agitation alters the dipole orientation of solvent molecules, weakening their coordination with Li⁺ and promoting the formation of an anion‐dominated solvation structure, which thereby significantly improves the cycling stability of batteries at high temperatures (Figure 4c). The cycling performance of the 1 Ah LiFePO_4_||Li pouch cell achieved a capacity retention rate as high as 93.54 % after 150 cycles at 1 C and 100 °C (Figure 4d). Moreover, benefiting from their inherent flame retardance, DEE‐based Li metal pouch cell successfully passed the nail penetration test without thermal runaway. These findings signify a shift in DEE research from performance optimization toward mechanism‐driven safety design.

### 4.3 Enhanced Safety

Thermal safety is a cornerstone for the application of high‐energy‐density LMBs. Traditional liquid electrolytes are mainly composed of flammable organic solvents with low flash point and high thermal sensitivity, which make them prone to decompose under overheating or abuse conditions.^[^ ^124^, ^125^ ^]^ These reactions release substantial heat and flammable gases, often leading to thermal runaway, fire, and even explosion.^[^ ^126^, ^127^ ^]^

**Figure 5:**
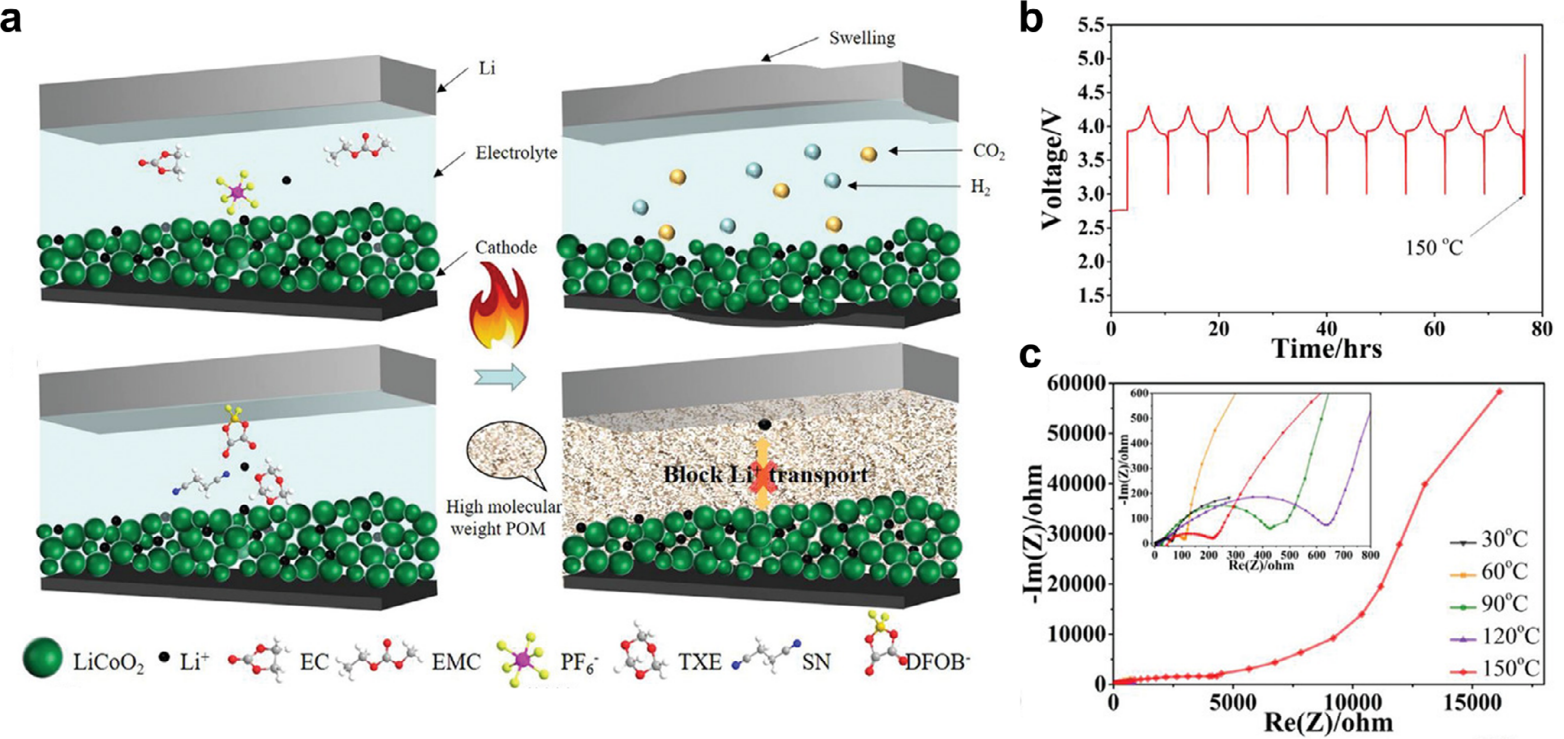
a) Thermal shut‐down function of LMBs using DEE: Schematic illustration and failure analysis of LMBs using carbonate‐based electrolyte and DEE. Reproduced with permission.^[^ ^81^ ^]^ Copyright 2022, Wiley‐VCH. b) Voltage profiles of a cell using DEE for 10 cycles at 25 °C, and then it was subjected to a thermal shock test (the temperature rises to 150 °C). Reproduced with permission.^[^ ^81^ ^]^ Copyright 2022, Wiley‐VCH.c) EIS results of the studied cells using DEE at different temperatures. Reproduced with permission.^[^ ^81^ ^]^ Copyright 2022, Wiley‐VCH.

DEEs show significant advantages in thermal safety due to their non‐volatility, flame retardance, and high thermal stability. Their thermal decomposition resistance across a wide temperature range helps inhibit the exothermic reaction with the electrode, delaying the thermal runaway process and considerably improving the battery thermal safety.^[^ ^128^ ^]^ Recently, Zhang et al.^[^ ^81^ ^]^ developed a thermally responsive DEE system that integrates rationally regulated solvation structure with an intelligent safety mechanism. This DEE electrolyte exhibits a thermal‐induced automatic shutdown function upon heating. Polymerizable monomers rapidly form high‐molecular‐weight polymers at high temperatures, causing the ionic conductivity to drop to near‐insulating levels and effectively terminating battery operation (**Figure** **5**a–c). This thermal‐induced shutdown mechanism transcends the limitations of conventional passive protection approaches of traditional liquid electrolytes, providing a new paradigm for intrinsically safe LMB design.

**Figure 6:**
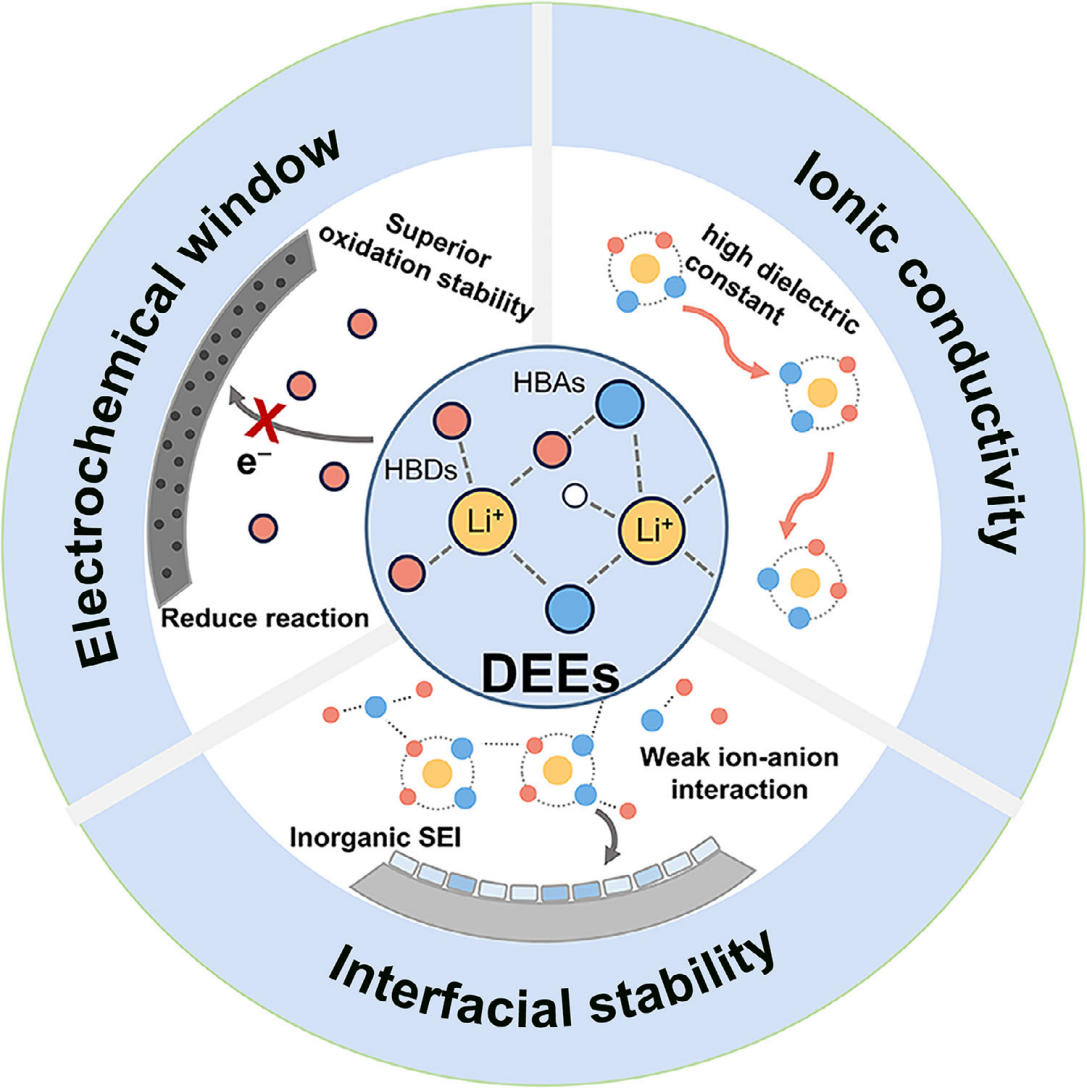
Schematic illustration of the solvation‐driven interfacial mechanism in DEEs.

In summary, the overall enhanced performance of DEE‐based batteries is attributed to unique solvation structures enabled by their HBD and HBA components (**Figure** **6**). On one hand, HBDs generally possesses high dielectric constant and superior oxidation stability, which not only facilitates Li^+^ transport kinetics but also improves the oxidation stability of DEEs. On the other hand, the moderate interaction between HBDs and HBAs promotes anion incorporation into the Li^+^ solvation sheath, leading to the formation of inorganic‐rich SEI on the Li metal anode, which significantly improves interfacial stability.^[^ ^129^, ^130^ ^]^ Future optimization efforts should focus on rational molecular structure design, multi‐eutectic systems, and functional additives to precisely engineer the solvation structure for high‐performance LMBs.

## 5 Application of DEEs in Solid‐State batteries

Current liquid electrolyte‐based LMBs generally suffer from inherent limitations such as safety risks, limited cycle life, and constrained energy density.^[^ ^131^, ^132^, ^133^, ^134^ ^]^ Moreover, the persistent issue of Li dendrite formation continues to hinder the practical deployment of high‐energy LMBs employing conventional liquid electrolytes.^[^ ^135^, ^136^, ^137^, ^138^ ^]^ To address these challenges, significant efforts have been directed toward the development of solid‐state electrolytes (SSEs), which represent a promising path to safer and more stable battery systems.^[^ ^139^, ^140^, ^141^, ^142^ ^]^ Solid polymer electrolytes (SPEs) as a particular class of SSEs have attracted considerable attention due to their unique advantages in manufacturing process, cost‐effectiveness, interface contact, and mechanical properties.^[^ ^143^, ^144^, ^145^, ^146^ ^]^ These characteristics make SPEs suitable for scalable manufacturing and various application scenarios.^[^ ^147^, ^148^, ^149^ ^]^ Nonetheless, the widespread adoption of SPEs is still hampered by several intrinsic drawbacks, including low room‐temperature ionic conductivity, narrow ESW, interfacial degradation, and insufficient safety under abusive conditions.^[^ ^150^, ^151^, ^152^, ^153^ ^]^

### 5.1 Enhanced Ion Transport

**Figure 7:**
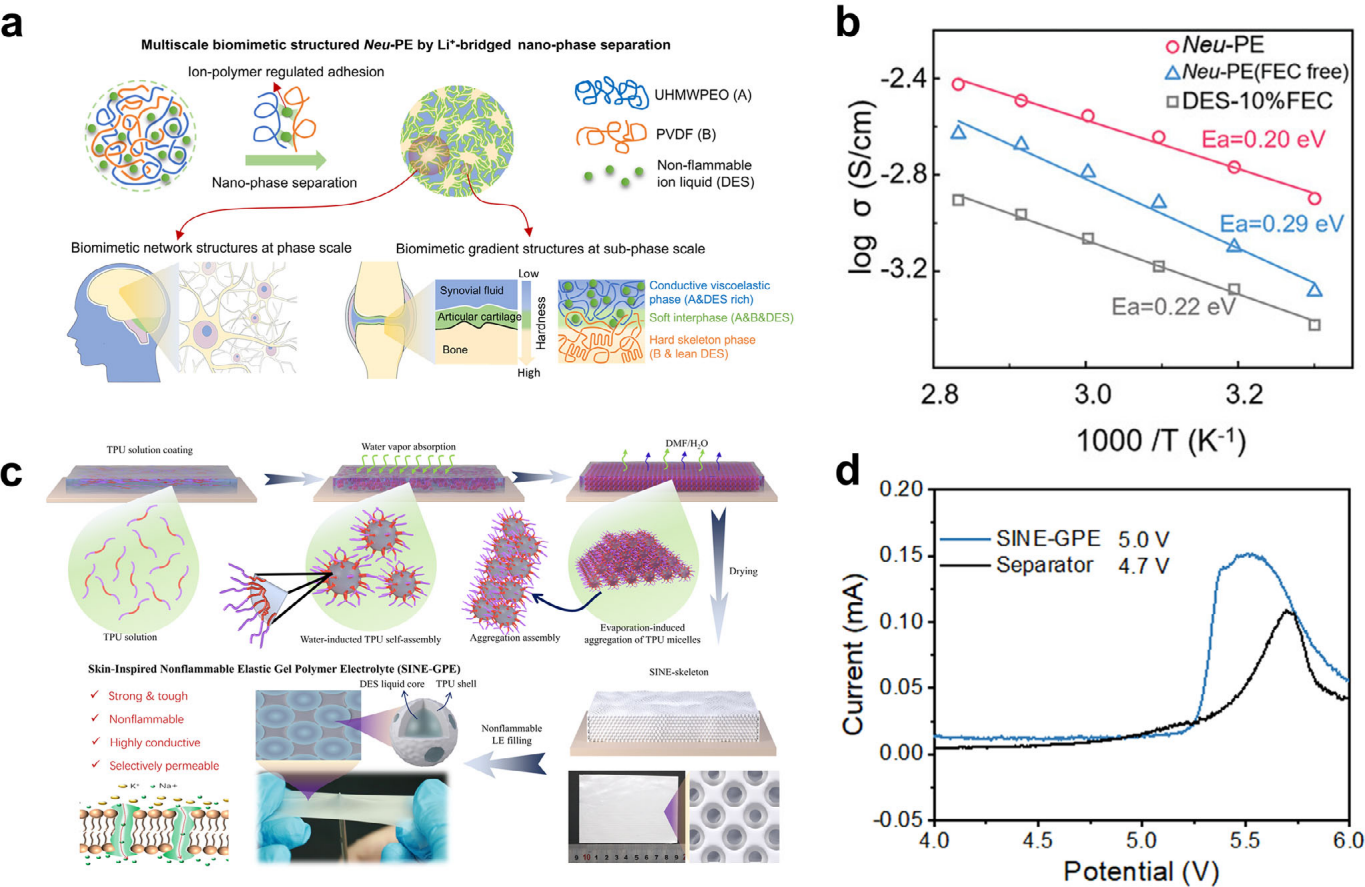
a) Design strategy of the multiscale biomimetic structure by Li^+^‐bridged nano‐phase separation. Reproduced with permission.^[^ ^157^ ^]^ Copyright 2023, Wiley‐VCH. b) Ionic conductivities as a function of temperature (30–80 °C). Reproduced with permission.^[^ ^157^ ^]^ Copyright 2023, Wiley‐VCH. c) Cellular‐pore formation mechanism of polymer framework and the preparation of gel polymer electrolytes by liquid encapsulation with flame retardance DEE. Reproduced with permission.^[^ ^158^ ^]^ Copyright 2025, Wiley‐VCH. d) The LSV curves of the gel polymer electrolytes as compared with commercial separators with DEE at 30 °C. Reproduced with permission.^[^ ^158^ ^]^ Copyright 2025, Wiley‐VCH.

As we all know, Li^+^ transport in SPEs strongly depends on the segmental motion of polymer chains. Liquid DEEs provide novel opportunities to overcome these limitations.^[^ ^154^, ^155^ ^]^ DEEs exhibit high ionic conductivity, flame retardance, and wide ESW comparable to that of traditional liquid electrolytes. When incorporated into SPEs, DEEs can not only promote the plasticization of polymer chains to facilitate Li^+^ transport but also enhance safety without compromising electrochemical performance.^[^ ^39^, ^156^ ^]^ Yang et al.^[^ ^157^ ^]^ utilized the Li^+^‐bridged molecular interactions between polymer chains to significantly increase the content of DEE encapsulated in the polymer matrix, thereby enhancing the ionic conductivity (**Figure** **7**a,b). Similarly, Li et al.^[^ ^158^ ^]^ discovered a strong interaction between the polymer framework and the DEE plasticizer, which effectively enhanced the electrochemical stability of the electrolyte, making it surpass that of any single component (Figure 7c,d).

### 5.2 Improved Interfacial Stability

**Figure 8:**
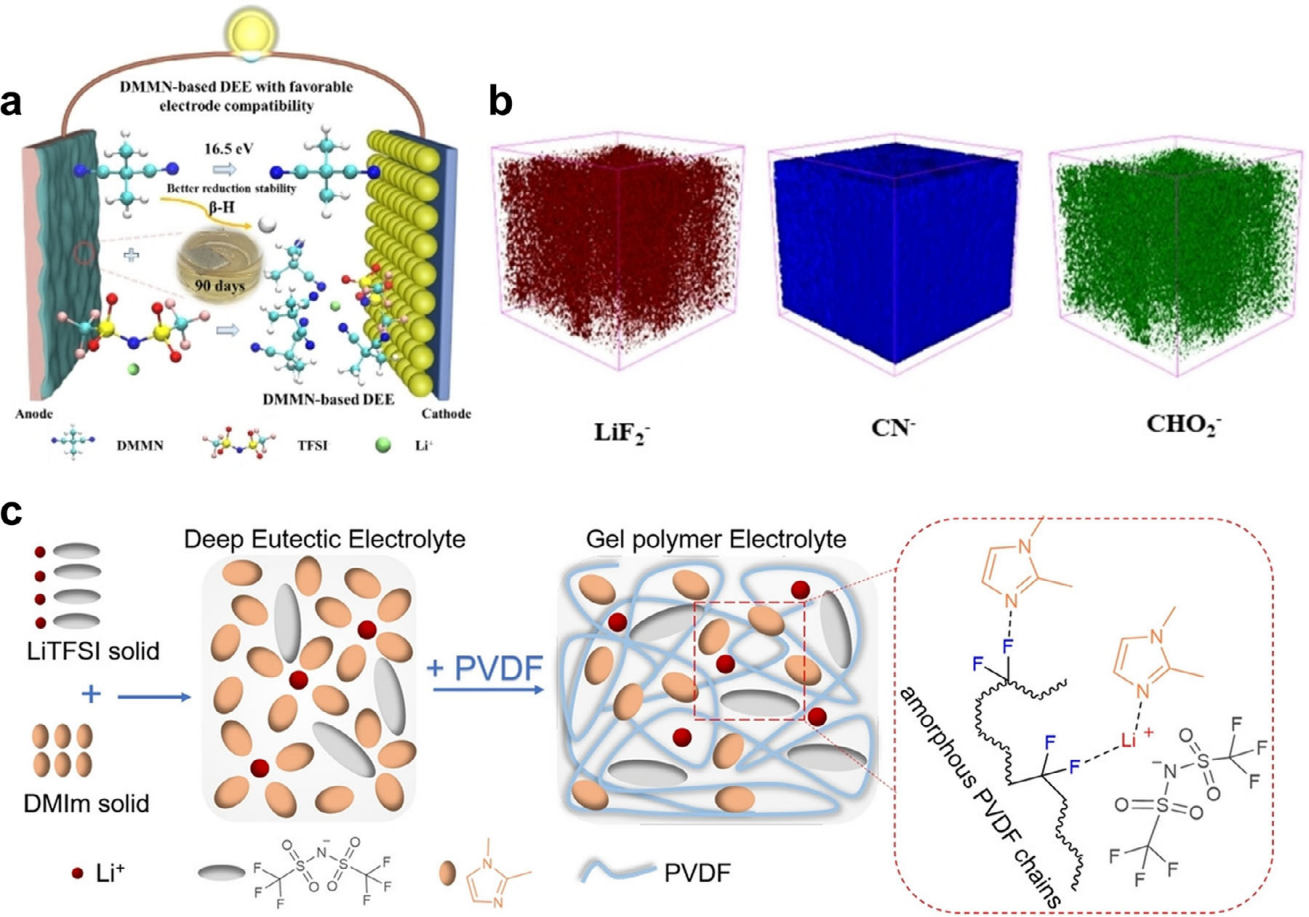
a) scheme of DEE with favorable electrode compatibility. Reproduced with permission.^[^ ^159^ ^]^ Copyright 2024, Wiley‐VCH. b) Time of flight secondary ion mass spectrometry negative ion depth profiles and compiled a 3D diagram of cycled Li anode harvested from the cycled Li||Li symmetric cells using DEE. Reproduced with permission.^[^ ^159^ ^]^ Copyright 2024, Wiley‐VCH. c) Diagram of the preparation process of DEE and DEE‐based gel polymer electrolytes. Reproduced with permission.^[^ ^160^ ^]^ Copyright 2022, Wiley‐VCH.

Interfacial instability represents another critical challenge for SPEs. Poor electrochemical compatibility often leads to undesirable side reactions at electrode interfaces.^[^ ^161^, ^162^, ^163^ ^]^ Currently, two main strategies have been employed to improve the interfacial stability using DEEs: molecular optimization of DEEs and synergistic design of DEE/polymer composites. For example, Zhao et al.^[^ ^159^ ^]^ significantly enhanced the reduction stability of electrolytes with Li metal by eliminating activated α‐H in DEE (**Figure** **8**a). Furthermore, in combination with fluoroethylene carbonate (FEC), a robust and elastic SEI was induced, thereby further enhancing the stability and cycling performance of the Li metal anode (Figure 8b). Alternatively, Pei et al.^[^ ^160^ ^]^ revealed that introducing electron‐withdrawing polymer chains into DEE altered the coordination environment of Li^+^ in DEE, induced the preferential decomposition of TFSI^−^ group on the Li metal surface, and constructed a LiF/Li_3_N‐rich SEI, thereby significantly improving the interfacial stability (Figure 8c). In addition, Lee et al.^[^ ^164^ ^]^ confined DEE within a polymer matrix to form nano‐domains, which suppressed interfacial side reactions and promoted uniform Li deposition. Moreover, as a fluid component in SPEs, DEEs facilitate the dissociation of Li salts and support the formation of a stable, protective interphase on Li metal, further improving the interfacial stability.^[^ ^165^ ^]^

### 5.3 Enhanced Safety

**Figure 9:**
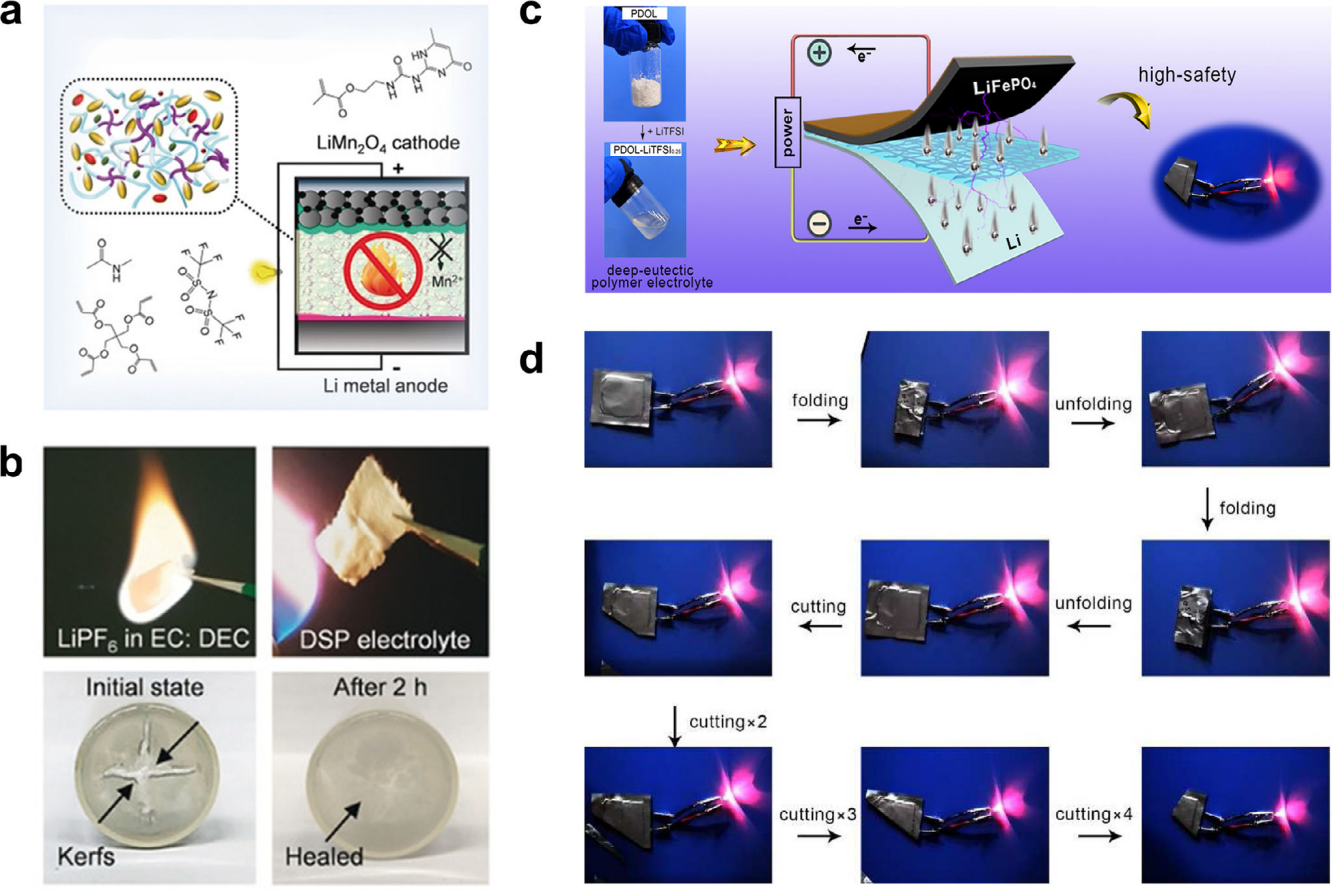
a) DEE‐based self‐healing polymer electrolytes. Reproduced with permission.^[^ ^166^ ^]^ Copyright 2020, Wiley‐VCH. b) Optical images of the 1 M LiPF_6_ in EC/DEC electrolyte (top left) and DEE (top right) under a combustion test, and the self‐healing process of the DEE after being cut (bottom). Reproduced with permission.^[^ ^166^ ^]^ Copyright 2020, Wiley‐VCH. c) Scheme of DEE‐based electrolytes preparation. Reproduced with permission.^[^ ^167^ ^]^ Copyright 2022, Elsevier. d) Safety test of battery at fully charged state. Reproduced with permission.^[^ ^167^ ^]^ Copyright 2022, Elsevier.

Safety remains a central concern for SPEs. Although generally less flammable than liquid electrolytes, some polymer matrices may still decompose under extreme conditions, releasing flammable gases and even causing thermal safety events.^[^ ^168^, ^169^ ^]^ DEEs possess high thermal stability and flame retardance, which provides an effective solution to address the aforementioned safety risks.^[^ ^170^ ^]^ For example, Jaumaux et al.^[^ ^166^ ^]^ revealed that the strong interaction between self‐healing polymers and DEE could effectively prevent the leakage of DEE. Combined with the excellent flame retardance of DEE, the safety of LMBs was significantly enhanced (**Figure** **9**a,b). Wang et al.^[^ ^167^ ^]^ directly mixed solid poly(1,3‐dioxolane) (PDOL) with solid LiTFSI. Driven by the strong hydrogen bond interactions between these two components, a DEE was directly formed (Figure 9c). The resulting SPE enabled a folded pouch cell to power a red LED and maintain operation even after multiple cutting tests (Figure 9d), highlighting exceptional mechanical and safety performance.

Despite the compelling safety features of DEEs‐based SPE, such as low volatility, high ionic conductivity, thermal stability, and flame retardance, they may still undergo degradation under extreme thermal, electrical, or mechanical abuse conditions, potentially leading to chain exothermic reactions and thermal runaway.^[^ ^171^, ^172^ ^]^ Therefore, relying solely on intrinsic material properties is insufficient to ensure safety under all realistic scenarios. Active safety mechanisms that enable real‐time monitoring and intervention are urgently needed to enhance the reliability throughout the battery lifecycle.^[^ ^173^, ^174^ ^]^

**Figure 10:**
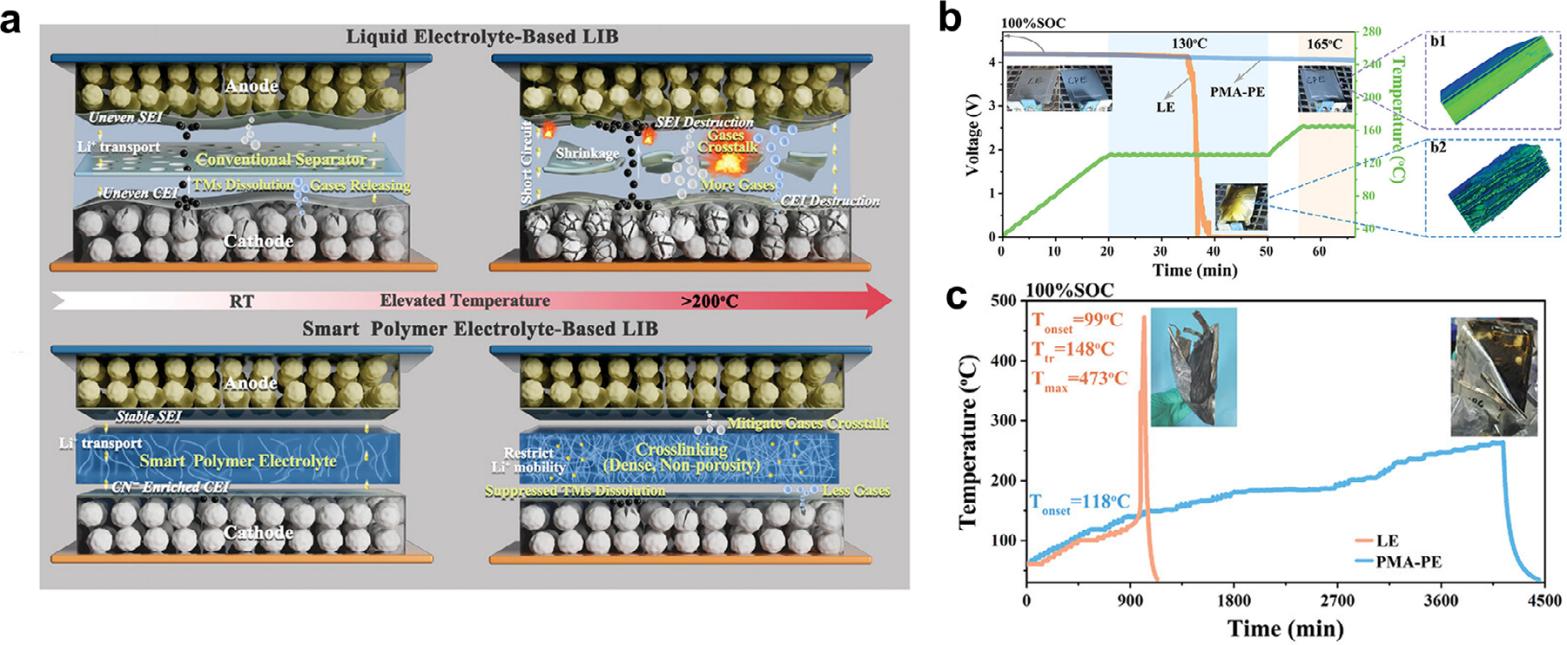
a) Schematic illustration of interelectrode crosstalk using the commercially available liquid electrolyte and schematic illustration of as‐prepared intelligent electrolytes with mitigating interelectrode crosstalk. Reproduced with permission.^[^ ^175^ ^]^ Copyright 2024, Wiley‐VCH. b) Temperature and voltage curves of pouch cells using LiPF_6_/EC‐DMC electrolyte and intelligent electrolytes under thermal abuse. The insets are the digital images of pouch cells before and after thermal abuse testing, and the 3D rendering images of pouch cells after thermal abuse obtained from CT measurements. Reproduced with permissionc.^[^ ^175^ ^]^ Copyright 2024, Wiley‐VCH. c) ARC testing of pouch cells using LiPF_6_/EC‐DMC electrolyte and intelligent electrolytes, and the insets are the digital images of pouch cells after the ARC testing. Reproduced with permission.^[^ ^175^ ^]^ Copyright 2024, Wiley‐VCH.

Toward this goal, Dong et al.^[^ ^175^ ^]^ developed a thermally responsive DEE system capable of spontaneous cross‐linking under high‐temperature conditions (**Figure** **10**a). This intelligent electrolyte effectively suppressed gas diffusion and transition metal dissolution, thereby mitigating thermal runaway risks. Surprisingly, Remarkably, it maintained thermal stability even at 250 °C (Figure 10b,c). Such intelligent polymer electrolytes with active safety protection features can rapidly respond to thermal threats, isolate electrodes, and halt propagation of runaway reactions. Combined with the inherent flame retardance of DEEs, they establish a dual safety barrier for LMBs, providing a strong guarantee for the future development of safe and sustainable energy storage systems.

## 6 Conclusion and Outlook

DEEs have attracted much attention in recent years and demonstrated considerable potential for applications in Lithium (Li) metal batteries (LMBs). Focusing on the interfacial stability, high‐temperature performance, and safety performance, this review provides a comprehensive analysis of the physicochemical properties of DEEs and their impact on Li battery performances. Compared to conventional liquid electrolytes, DEEs demonstrates remarkable properties, including high ionic conductivity, excellent thermal stability, flame retardance, and a wide ESW. They significantly contribute to improved safety and electrochemical performance in both liquid and solid‐state LMBs. Despite these advantages, the practical application of DEEs remains critical challenges. A primary concern is the poor compatibility between acidic α‐hydrogen in DEEs and the Li metal anode, which triggers detrimental side reactions at the electrode/electrolyte interface. Furthermore, the extensive hydrogen bond network endows DEEs with high viscosity, consequently limiting Li⁺ transport kinetics and overall battery performance.^[^ ^176^, ^177^ ^]^ Therefore, in‐depth research on DEEs is therefore essential for enhancing electrochemical performance, battery safety, and mitigating fire risks.

Given the pivotal role of DEEs in next‐generation LMBs, the following potential research directions were proposed:

1. The polymerized deep eutectic electrolytes (PDEEs) are derived from DEEs by introducing a polymerizable functional group (e.g., double bonds and epoxy groups), enabling in situ polymerization under specific conditions to form a structured polymer network. This approach combines the advantageous features of DEEs with the flexibility and processability of polymers, significantly enhancing both electrochemical performance and safety of batteries. Future work should focus on designing novel PDEE chemistries that possess high ionic conductivity while maintaining interfacial stability.
2. Recently, a specific non‐covalent interaction involving Li⁺ and electron‐donating atoms or π‐systems plays a critical role in the formation and stabilization of DEEs. This interaction strongly influences the eutectic behavior and electrolyte structure, which thereby facilitates the ionic conduction of Li⁺ and the formation of stable electrode/electrolyte interfaces. Given the importance of this interaction in regulating ion transport and interfacial stability, in‐depth understanding of DEE formation mechanism should be further emphasized, which can further expand the applicability of DEEs and provide new foundational strategies for building high‐performance battery systems.
3. Artificial Intelligence (AI) is profoundly changing the research paradigm of materials science by overcoming the limitations of traditional trial‐and‐error approaches. Through data mining, machine learning, and automated experimentation, AI can drastically reduce the development timeline for new materials while improving prediction accuracy for complex properties. By leveraging AI‐driven tools, researchers can efficiently screen hydrogen bond acceptors (HBAs) and donors (HBDs), accelerating the discovery of DEE systems tailored for high energy density, long cycle life, and enhanced safety. This approach may provide breakthrough solutions to critical challenges in battery cost, performance, and safety.

With growing scientific interest and increasing research investment in DEEs, it is reasonable to anticipate substantial progress in key performance metrics such as energy density, cycle life, and safety in the near future. DEEs are poised to inject new vitality into the innovation of Li battery technology and promote its widespread application in the renewable energy field.

## Conflict of Interest

The authors declare no conflict of interest.
